# J. B. Killock

**Published:** 1892-02

**Authors:** 


### J. B. HILLOCK.

J. B. Hillock, V. S., Lancaster, Ohio, died July 26th, 1891.

Dr. Hillock was a member of the Ohio Association from its origin and always took an active interest in its welfare. His death was exceptionally sad from the fact that it was unexpected, and brought about in the course of his professional duty, dying of acute septicæmia. On Tuesday, July 21, he held a post-mortem on an animal that died of pneumonia, slightly cutting the knuckle of the second finger of his right hand. It was a very slight injury and no attention whatever was paid to it, until Thursday, when it began to cause some pain, but not sufficient as to regard it at all serious. Meeting his physician he consulted him, who advised treatment, which was followed, still attending to his daily practice, which he continued to do up till Saturday night, leaving his office for home at 9 P. M.

He did not complain, and rested fairly well during the night and Sunday morning, although not feeling well, he concluded to attend church. Just at this time his physician was passing and called in and advised him not to go out, so he remained at home. In a very short time he rapidly grew worse, having slight convulsions and heart failure, he became unconscious about noon, remaining so until his death, which occurred the same day at 7:45 P. M.

Resolutions.—*Whereas*, In the prime of life and in the course of duty death has removed our colleague and associate, Dr. J. B. Hillock, and

*Whereas*, Our professional and business relations with him, were at all times pleasant. Therefore :

*Resolved*, That in his death we mourn the loss of a friend and valued member of the profession and this Association, and one who had merited our highest respect and esteem, and

*Resolved*, That our heartfelt sympathies be extended to his relations and friends, with the hope that even such a loss may be overruled for greater good, and

*Resolved*, That copies of these resolutions be sent to the family and to the veterinary journals, and also spread upon the minutes of this Association.

J. D. Fair.<br>

T. B. Cotton. } Committee.<br>

W. E. Weight.

